# The Therapeutics of Music

**Published:** 1904-08

**Authors:** 


### EDITORIAL NOTES AND COMMENTS.

The Business office of THE JOURNAL-RECORD is No. 1007-1008 Century Building.

The Editorial office is 318-319-320 The Prudential.

Address all Business communications to Dr. M. B. Hutchins, Mgr.

Make remittances payable to THE ATLANTA JOURNAL-RECORD OF MEDICINE.

On matters pertaining to the Editorial and Original Communications address Dr. Bernard Wolff, Atlanta.

Reprints of original articles will be furnished at cost price. Requests for the same should always be made *on the manuscript*.

We will present, post-paid, on request, to each contributor of an original article, twenty (20) marked copies of THE JOURNAL-RECORD containing such article.

### THE THERAPEUTICS OF MUSIC.

*Quand on perd par triste occurrence*
*Sou espèrance et sa gaieté,*
*La remède au mèlancholique*
*C'est la musique et la beauté.*

—*De Musset.*

SOME time ago it was the writer's melancholy duty to attend a funeral. The circumstances surrounding the death were peculiarly sad. The varying facial expressions of the bereaved family could be followed through the preacher's discourse, on to the funeral songs. When the speaker referred to the notable virtues of the deceased, the expressions indicated profound grief but with the emotions controlled, yet when the sweet strains of the organ and the tender hymn stole forth lamentations burst out without effort at repression. The thought came to mind that the powerful influence wrought upon the emotions by music under these conditions would be equally effective in other disordered states of the nervous system, and lent credence to the serious suggestion offered from time to time of its deliberate utilization therapeutically. Instability of the nervous system manifested by various psychoses may as well be treated by music of an appropriate kind as by drugs, for the effects are similar, whether produced by harmonious sounds or by drugs.

Hashish is aphrodisiac in the visions conjured up by it and an Offenbachian lyric brings forth images of the glittering row of swaying coryphees with the symmetrical central figure transmuting penseroso to allegro like a trick of legerdemain, and makes the heart sing a pæan of praise that youth is not all flown. The "Schlummerlied" is equal to a dose of trional and an inferno fugue of Bach will drive sleep away like a drink of coca-cola.

The roll of the drum and the bubbling piccolo will bring a scornful smile to the lips at the idea of death in the same pot-valiant manner that would make Falstaff as hardy as a Nemean lion.

But the addition of music to the therapeutic armamentarium would be fraught with difficulties, especially in properly carrying out the prescription. The spectral screeching of the phonograph would be an injurious method of administration, yet its use suggests itself. Not every doctor is a skilled performer on the harmonica or the jews-harp, though such an instrument is readily portable and would occupy not more space than a pocket-comb, which in itself backed by a slip of paper offers limited musical possibilities. The transferring to a patient's residence of a melodeon or a piano might sometimes be embarrassing—would be, in fact, unless the doctor's buggy was built on the scheme of a furniture van, a questionable subordination of beauty to utility.

These objections are too cogent to be overcome in miscellaneous practice, but the suggestion of adding a carefully edited repertoire of music to the sanitarium idea is thrown out for what it is worth to those captains of industry who are the possessors of such establishments.

